# Sensory neurons: unveiling the symphony of wound healing

**DOI:** 10.1038/s41392-024-01880-7

**Published:** 2024-07-24

**Authors:** Dongsheng Jiang, Hans-Günther Machens, Yuval Rinkevich

**Affiliations:** 1 Precision Research Center for Refractory Diseases, Shanghai General Hospital, Shanghai Jiao Tong University School of Medicine, Shanghai, China; 2 Trauma Medical Center, Shanghai General Hospital, Shanghai Jiao Tong University School of Medicine, Shanghai, China; 3 Department of Plastic and Hand Surgery, Klinikum rechts der Isar, School of Medicine, Technical University of Munich, Munich, Germany; 4 Institute of Regenerative Biology and Medicine, Helmholtz Center Munich, Munich, Germany

**Keywords:** Peripheral nervous system, Innate immune cells, Trauma

In a recent paper published in *Nature*, Lu et al.^1^ reveal a pivotal role of Na_v_1.8^+^ nociceptor-released neuropeptide calcitonin gene-related peptide (CGRP) in modulating the activities of myeloid cells during skin and muscle injury repair. This study demonstrated the significant impact of sensory neurons on injury-induced inflammation and wound healing.

Tissue injury and subsequent repair pose substantial clinical challenges in the form of chronic wounds or fibrotic scarring. In such clinical circumstances, wound healing does not progress through the normal sequential phases of hemostasis, inflammation, proliferation and remodeling. Specifically, dysregulation of myeloid cells (monocytes, macrophages, neutrophils) is directly associated with various clinical forms of maladaptive wound healing, ranging from non-healing wounds to tissue fibrosis and excessive scarring.

Although the peripheral nervous system has not received as much attention relative to endothelial/vascular and immune cell components, the role of peripheral nerves in tissue repair has not been entirely neglected. Studies conducted in animal models over 60 years ago, involving experimental resection of peripheral nerves, have demonstrated a connection between nerve endings and skin wound healing.^2^ Not only neurons, but also peripheral glial cells such as Schwann cells are instrumental in wound healing by undergoing de-differentiation, proliferation, and releasing pro-healing paracrine factors, such as transforming growth factor-β.^3^

Lu et al.^1^ uncovers an additional critical role of the nerve endings specifically tasked with sensing pain (Na_v_1.8^+^ nociceptors) in influencing myeloid cells during tissue injury. Nociceptors are specialized sensory neurons tasked with pain perception, and they enable the organism to detect inflammation, extreme temperature changes, and mechanical pressure. Leveraging *Nav1.8*^*cre*^*;Rosa26*^*DTA*^ double-transgenic mice, the authors were able to selectively deplete Na_V_1.8^+^ dorsal root ganglion neurons via diphtheria toxin-mediated cell death, and employed two injury models: dorsal skin full-thickness wounds and upper thigh quadriceps muscle loss. Their findings in rodents demonstrate that the absence of Na_V_1.8 nociceptors significantly delay skin wound closure and diminished muscle regeneration while increasing fibrotic scars.

**Fig. 1:**
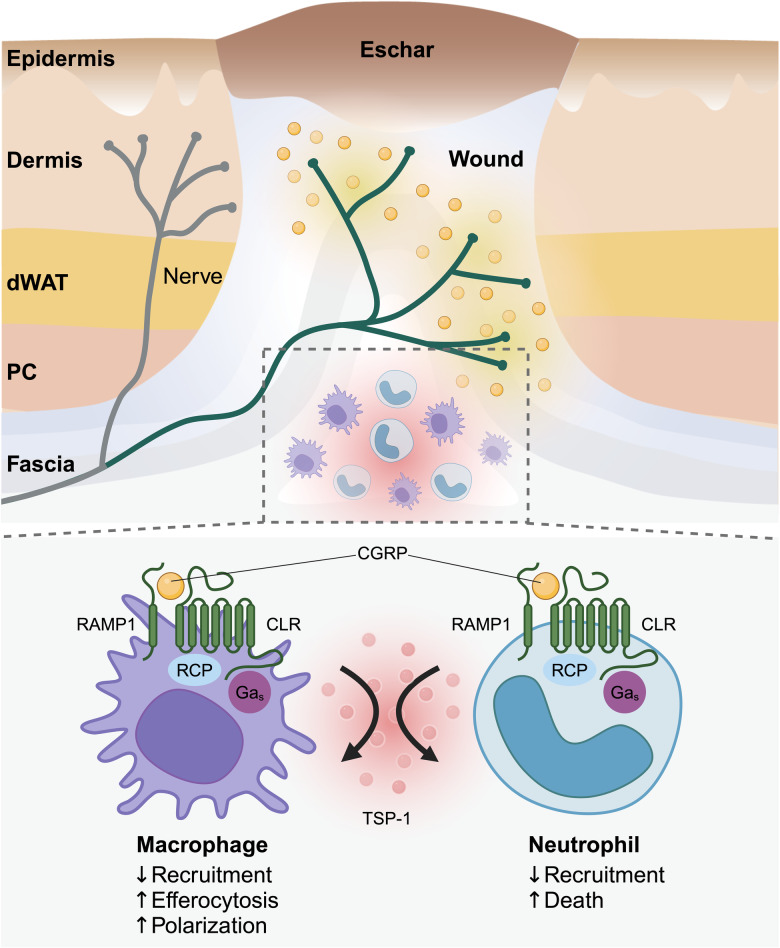
Nociceptor endings extend into wounds, releasing the neuropeptide CGRP, which regulates the activities of wound infiltrated neutrophils and macrophages. CGRP inhibits the recruitment of neutrophils and macrophages towards chemokines commonly found in wounds (CXCL1 for neutrophils and CCL2 for macrophages). Additionally, CGRP triggers neutrophil and macrophage cell death in the presence of proinflammatory cytokines (IL-1β and TNF). CGRP enhances macrophage efferocytosis both in the absence and presence of inflammatory cytokines. Furthermore, CGRP accelerates macrophage polarization toward a pro-repair phenotype in the presence of anti-inflammatory cytokines (IL-4 + IL-13 or IL-10). These effects are further amplified by the autocrine/paracrine release of TSP-1 from these myeloid cells, facilitating wound healing. dWAT dermal white adipose tissue, PC panniculus carnosus, CGRP calcitonin gene-related peptide, RAMP1 receptor activity-modifying protein, CLR calcitonin-like receptor, RCP receptor component protein, TSP-1 thrombospondin-1. (Created with BioRender.com)

Employing *Nav1.8*^*cre*^*;Rosa26*^*tdT*^ mice to track nociceptors with red fluorescence protein, the researchers pinpointed that Na_V_1.8^+^ neurons were activated upon tissue injury, and nerve endings extend into the granulation tissue formed after skin and muscle injuries, releasing a neuropeptide called CGRP. Notably, non-activated nociceptors do not secret CGRP during homeostatic non-injured conditions. CGRP acts as a ligand for receptor activity modifying protein (RAMP1) expressed on myeloid cells. Mice with reconstituted bone marrow cells deficient in RAMP1 exhibit severe impairment in skin and muscle healing, mirroring the effects observed in Na_V_1.8 nociceptor-ablated mice. Furthermore, the authors confirmed that sensory neuron-derived CGRP promoted tissue healing by directly modulating myeloid cells. This was verified in *LysM*^*cre*^*;Ramp1*^*fl/fl*^ mice, where LysM^+^ myeloid cells lack RAMP1 expression. Strikingly, these mice exhibited similar healing impairment as observed in nociceptor-deletion models. Depletion of nociceptors in *Nav1.8*^*cre*^*;Rosa26*^*DTA*^ mice led to elevated numbers of neutrophils and pro-inflammatory macrophages in the injury sites, and to delayed wound healing. Through in vitro assays and an adoptive transfer model with *Ramp1*^*-/*-^ myeloid cells, the authors further demonstrated that CGRP signaling regulated myeloid cell dynamics in many ways, including reducing myeloid cell recruitment and augmenting cell death, enhancing clearance of debris/dead cells by efferocytosis, and promoting macrophage polarization needed for wound healing to progress. These effects were shown to be further amplified by the autocrine and paracrine release of thrombospondin-1 by myeloid cells (summarized in Fig. 1).

Leveraging these findings linking nociceptor-CGRP to inflammation, the authors developed a pre-clinical approach that ensures sustained release of CGRP at the wound site by fusing CGRP with an ECM-binding sequence (eCGRP), followed by a plasmin-sensitive sequence to facilitate retention and gradual release of CGRP at the injury site. Application of eCGRP, either topically to wounds or via injection into muscle using a fibrin hydrogel, successfully rescued the healing deficits observed in nociceptor-depleted *Nav1.8*^*cre*^*;Rosa26*^*DTA*^ mice. Finally, the efficacy of eCGRP was validated in a clinically relevant db/db mice, a leptin receptor-deficient mouse model that exhibits type 2 diabetes with peripheral neuropathy. Treatment with eCGRP markedly improved wound closure and muscle regeneration in the diabetic setting.

Recent research has uncovered additional critical roles of sensory neurons in immune regulation. For instance, Hanc et al. demonstrated that nociceptors can modulate dendritic cell functions through various mechanisms to maintain skin barrier function, including the release of neuropeptide CGRP and chemokine CCL2, as well as depolarization of DC cell membranes via calcium fluxes.^4^ Similarly, in the lung models of inflammation, vagal sensory neurons release CGRPβ via JAK1 activation, which can suppress the function of group 2 innate lymphoid cells and mitigate allergic inflammation.^5^

Clinical indications further illustrate the link between neuro-inflammation and wound healing. For example, peripheral neuropathies pose significant challenges to skin wound healing, notably exemplified in conditions such as diabetic foot ulcers (DFUs). Clinical classification of DFUs often revolves around symptoms of neuropathies and vascular pathologies. While the mechanistic and therapeutic aspects of vascular pathologies with deficient circulation have been extensively explored, progress in understanding the impact of neuropathies on DFUs has been slow. Further links between neuro-inflammation and wound healing are also evident in other clinical scenarios. For example, it is widely recognized, albeit often unreported, that paraplegic patients experience markedly delayed wound healing. A second example lies in current clinical practice, where the delaying effect of denervation on skin incision healing is demonstrated in cutaneous wounds involving fasciocutaneous- or myocutaneous-free microvascular tissue. In these cases, autologous tissue containing skin, blood vessels, and nerve fibers is surgically excised and microsurgically transplanted into tissue defects following tumor resection or trauma, without the reconnection of concomitant sensory nerve fibers.

Therefore, innovative approaches that bypass or reactivate the neuro-immune axis in wounds, such as with eCGRP presented in Lu et al., holds promise for advancing therapeutic strategies in the context of impaired wound healing across a range of clinical settings.
